# HIV Sexual Transmission Is Predominantly Driven by Single Individuals Rather than Discordant Couples: A Model-Based Approach

**DOI:** 10.1371/journal.pone.0082906

**Published:** 2013-12-20

**Authors:** David Champredon, Steve Bellan, Jonathan Dushoff

**Affiliations:** 1 School of Computational Science and Engineering, McMaster University, Hamilton, Canada; 2 Center for Computational Biology and Bioinformatics, University of Texas at Austin, Austin, Texas, United States of America; 3 Department of Biology, McMaster University, Hamilton, Canada; Fundacion Huesped, Argentina

## Abstract

Understanding the relative contribution to HIV transmission from different social groups is important for public-health policy. Information about the importance of stable serodiscordant couples (when one partner is infected but not the other) relative to contacts outside of stable partnerships in spreading disease can aid in designing and targeting interventions. However, the overall importance of within-couple transmission, and the determinants and correlates of this importance, are not well understood. Here, we explore how mechanistic factors – like partnership dynamics and rates of extra-couple transmission – affect various routes of transmission, using a compartmental model with parameters based on estimates from Sub-Saharan Africa. Under our assumptions, when sampling model parameters within a realistic range, we find that infection of uncoupled individuals is usually the predominant route (median 0.62, 2.5%–97.5% quantiles: 0.26–0.88), while transmission within discordant couples is usually important, but rarely represents the majority of transmissions (median 0.33, 2.5%–97.5% quantiles: 0.10–0.67). We find a strong correlation between long-term HIV prevalence and the contact rate of uncoupled individuals, implying that this rate may be a key driver of HIV prevalence. For a given level of prevalence, we find a negative correlation between the proportion of discordant couples and the within-couple transmission rate, indicating that low discordance in a population may reflect a relatively high rate of within-couple transmission. Transmission within or outside couples and among uncoupled individuals are all likely to be important in sustaining heterosexual HIV transmission in Sub-Saharan Africa. Hence, intervention policies should be broadly targeted when practical.

## Introduction

Diseases spread by sexual intercourse can be transmitted through a wide variety of social routes: within a stable, monogamous relationship; within a stable, non-monogamous relationship; or in casual encounters between people who may or may not also be involved in stable relationships. Understanding the importance of these routes for disease spread is important for making predictions and designing public-health interventions. Recent debates about HIV control have involved discussion of the importance of stable, “serodiscordant” partnerships (partnerships where one partner is infected and the other is not) to disease transmission [1]–[10].

Serodiscordant couples can arise from extra-couple transmission, or from new pairings involving a person who was infected either while single, while in a previous relationship or, more rarely in Sub-Saharan Africa, via non-sexual transmission (e.g. injection drug use, blood transfusions, or vertical transmission). Similarly, serodiscordant couples can be “lost” through couple dissolution, infection of the seronegative partner via either within-couple or extra-couple transmission, or the death of a partner via AIDS-related or unrelated causes. Serodiscordant couples represent a clear example of an individual at risk for transmission, and a valuable lens through which to study transmission risk and evaluate interventions [11]. If most transmission occurs within stable, serodiscordant couples, then couple-based intervention is a promising route for cost-effective interventions. However, if a lot of transmission is occurring outside of couples, population-based interventions will be necessary.

The relationship between the number of serodiscordant couples in a population and their role in transmission is complicated. Looking *forward* in time, the presence of serodiscordant couples implies potential risk of within-couple transmission in those very couples. Conversely, looking *backward* in time, the presence of serodiscordant couples implies that the infected individual was infected by somebody other than the current partner, and thus implies an increased importance of non-couple routes of transmission or of partner switching.

Dunkle *et al.* [2] used a “forward” approach to suggest that transmission between partners in serodiscordant couples contributed to the majority of all new HIV infections. In a follow-up study, Coburn *et al.* [5] used a similar forward approach to argue that transmission within stable serodiscordant couples can be an important driver of the HIV epidemic when the proportion of coupled individuals in a population is large. Importantly, such “forward” modelling directly considers the potential *contributions* of serodiscordant couples to new HIV incidence, but not their *origin*.

In the “backward” approach, inference is based instead on the origin of serodiscordance. A high level of serodiscordance is thus seen as evidence of outside infection. Such studies ([1], [3], [4], [12]) have concluded that within-couple transmission plays a smaller role in contributing to HIV incidence than Dunkle *et al.* [2]. For example, Lurie *et al.* [4] investigated serodiscordance through a specific group of migrant populations in rural South-Africa and estimated that a migrant man living in a stable couple was 26 times more likely to be infected outside this partnership rather than within. More recently, Bellan *et al.* [7] fitted a mechanistic model to Demographic and Health Surveys (DHS) data from several countries in Sub-Saharan Africa that combined both the “forward” and “backward” approaches and concluded that within-couple, pre-couple and extra-couple transmission are all important in most of the countries considered.

Some studies have looked specifically at within- versus extra-couple transmission within serodiscordant couples [1], [9], [13]. For example, Chemaitelly *et al.* [9] concluded that extra-couple infections contribute “minimally” to HIV incidence within serodiscordant couples in Sub-Saharan Africa, especially in countries with low overall HIV prevalence. Extra-couple transmissions has also been suspected to drive the number of serodiscordant couples [1]. Serodiscordant couple cohort studies have additionally found that 13–32% of seroconversions in seronegative partners were not virologically linked to their partner’s virus and thus due to extra-couple infection [14]–[18]. However, couples in cohorts may not be representative of the general population, are HIV serostatus-aware, and heavily counselled with resulting effects on their behavior [18].

The epidemiological role of serodiscordant couples changes throughout the course of an epidemic [3], [8], [11], and its evolution over time is complex. Robinson *et al.* [8] used individual-based simulations fitted to data from rural Uganda to conclude that within-couple transmission was the main route of infection once the HIV epidemic reaches an endemic phase. Johnson *et al.* [6], on the other hand, fitted a Bayesian model to prevalence and sexual-behaviour data in South-Africa, and concluded that HIV incidence continues to result predominantly from transmission outside of stable relationships.

The studies discussed above all focus on the amount of transmission that occurs directly through various routes. Direct transmission is clearly relevant, but is not the only factor determining the importance of a route. Some routes of transmission may be disproportionately important in spreading infection throughout the population. To take an extreme example, the amount of direct transmission of immunodeficiency viruses from non-humans to humans is negligible; but without early transmission through that route, there would have been no HIV epidemic. Here we take a complementary approach to earlier studies that focus on routes of transmission by using a simple dynamic model that allows us to ask not only what factors affect the amount of transmission through various routes, but also how changing transmission rates along various routes is expected to affect long-term disease prevalence.

We construct a partnership-based model specifically aimed at comparing the effects of transmission within stable couples, transmission to and from uncoupled individuals, and “extra-couple” transmission to and from coupled individuals. Partnership-based models have previously been used to study various aspects of sexually transmitted infections (STI) (see [19] for a recent review). Many of these trace back to the work of Dietz and Haldeler [20], who used a simple model to gain analytic insight into a model with sequential partnerships. Although previous dynamical models involving pair formation have been used to study various issues associated with the spread of STIs, no dynamical model has focused specifically on the contribution of transmission within serodiscordant couples to HIV incidence and prevalence. We explore the behaviour of our model across a range of parameters representative of HIV in Sub-Saharan Africa using latin hypercube sampling.

## Materials and Methods

### Model Formulation

Many of the parameters involved in modelling both couple formation and disease transmission are difficult to estimate, since they relate to private behaviours associated with strong social expectations. We therefore made this model as simple as seemed reasonable in order to disentangle and interpret the fundamental mechanisms involved. Our model explores the role of serodiscordance and within-couple transmission in HIV spread. In particular, we do not model genders separately. Including gender in the model would add a lot of complexity (and parameters), and is not necessary for addressing our question, since evidence suggests that the gender-specific proportion of index cases [7], [21] and probabilities of transmission [22] are at least roughly similar. Nor do we account for stages of HIV infectiousness, circumcision, co-infections or condom use.

We do include individual heterogeneous infection risk by phenomenologically reducing the contact rate as disease prevalence increases. This is a common method for introducing heterogeneity into transmission models without substantially increasing model complexity [23]. In particular, it allows the model to capture the early rapid rise in prevalence with realistic parameters and long-term behaviour. While we allow for extra-couple transmission by coupled individuals (i.e. once-off contacts while in a stable relationship), we do not keep track of more than one *stable* partnership per individual – a form of “concurrency” that is potentially important to HIV spread [24].

### Model Structure

**Figure 1:**
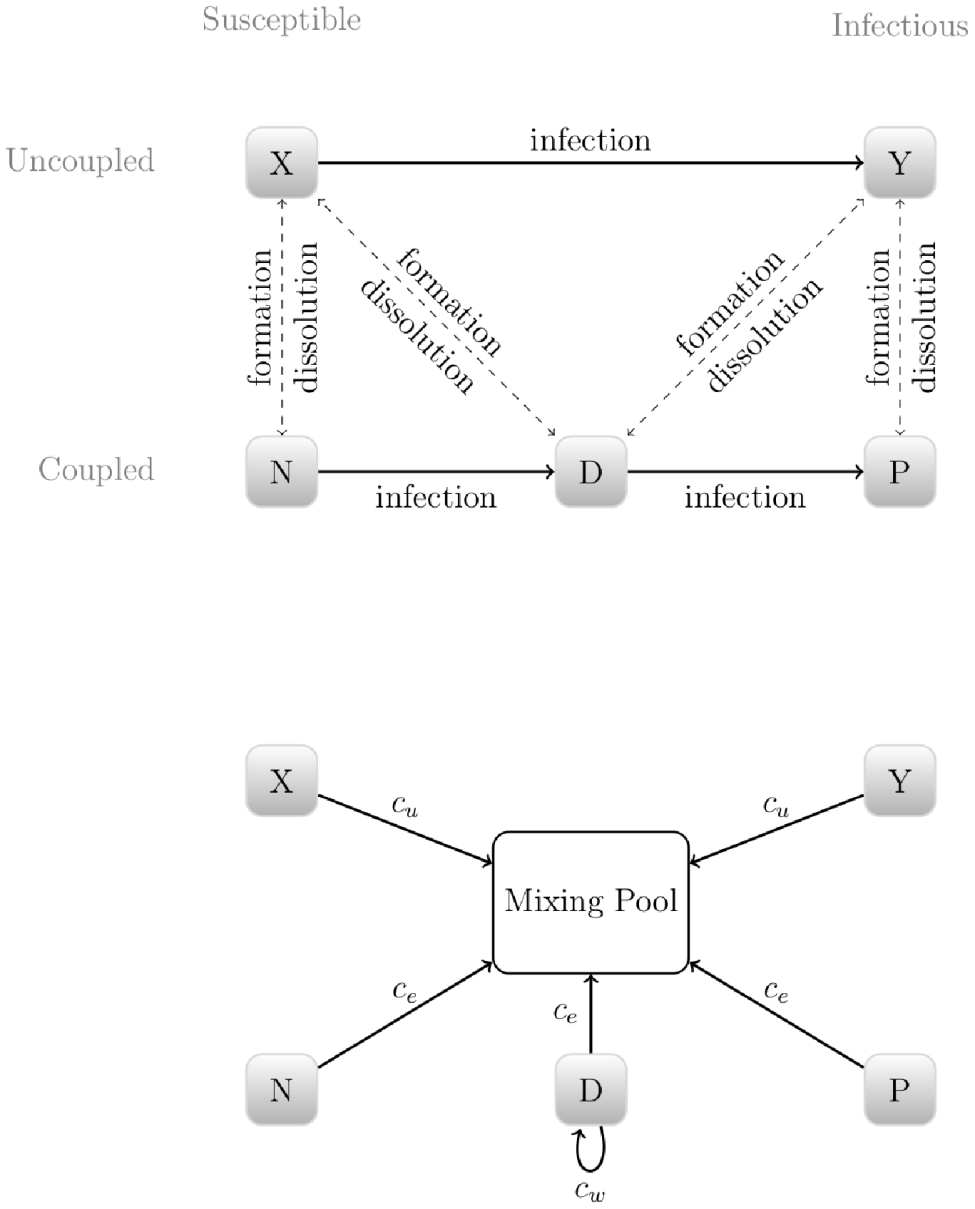
Model diagram. The top panel describes all possible movements between compartments. The bottom panel shows the infection pathways for each group. The mixing pool is an abstract representation of where all extra-couple sexual contacts occur.

We model uncoupled individuals and couples, classified by HIV status. Uncoupled susceptible individuals are denoted 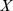 and uncoupled infectious individuals are denoted 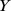. Couples are classified as 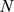 (concordant **n**egative) when both partners are susceptible; 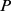 (concordant **p**ositive) when both partners are infectious; and 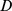 (sero**d**iscordant) when only one partner is infectious. The total number of individuals at any given time is 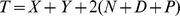 and the total number of infectious individuals is 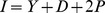. See Figure 1 for a graphical representation.

We assume that individuals die naturally at rate 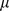 and that new individuals are recruited into the sexually active population as uncoupled susceptibles (compartment 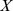) at rate 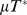 (thus, 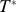 is the equilibrium population size in the absence of disease). Uncoupled individuals form couples at rate 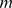 and couples dissolve at rate 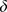. Infected individuals die of AIDS at rate 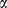. Marital parameters 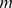 and 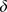 do not depend on infectious status.

Extra-couple intercourse is modelled by allowing both individuals in stable couples and uncoupled individuals to interact in a general mixing pool. Coupled and uncoupled individuals participate in this abstract pool at different rates, but they mix freely and proportionally in the pool. This allows us to keep the model simple and the number of parameters limited, while allowing for both partnership dynamics and the effects of extra-couple transmission on epidemic dynamics. Note that we formally model the short-term relationships as “one offs”, but our interpretation is intended to cover all but the main partnership. This is a substantial simplification, but not at all rare: in fact, many influential models implicitly treat all relationships as one off [19].

### Couple Formation and Dissolution

The size of the uncoupled population is 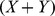, so partnerships are formed at total rate 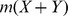. Since we assume that individual behaviour towards couple formation or dissolution is unaffected by infection status, the proportion of new couples for each type will follow a binomial distribution (see File S1 for more details):

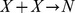
: 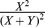

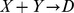
: 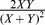

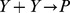
: 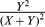

Each of these proportions is multiplied by the total rate 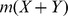.

The dissolution dynamics for coupled individuals is straightforward: 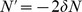, 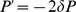 and 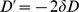. After dissolution, only the susceptible partner of 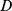 moves to 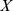 and both partners of 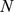 moves to 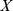, hence 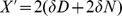. Similarly, only the infected partner of 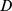 moves to 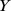 and both partners of 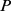 moves to 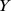: 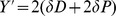.

Thus, we can write the effects of only couple formation and dissolution on the dynamics:
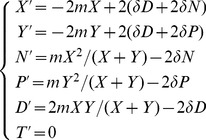
(1)


### Transmission

Susceptible individuals in serodiscordant couples become infected at the **w**ithin-couple effective mixing rate 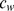 (individuals in seroconcordant couples are implicitly assumed to experience the same mixing rate, but do not transmit infection to each other). We also assume that coupled individuals mix with individuals outside the relationship with an **e**xtra-couple effective mixing rate 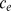, and thus become infected (if susceptible) at rate 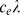, where 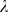 is the proportion of their contacts that are infectious. Similarly, **u**ncoupled individuals are exposed at rate 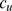 and become infected at rate 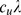.

The “effective mixing rates” 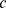 thus represent the rate at which individuals become infected through various routes, conditional on their partners being infectious. All of our mixing rates are best considered as effective mixing rates that combine frequency of contact and rate of partner change (for 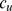 and 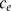 only). They implicitly aggregate all other effects important for transmission (like condom use, circumcision, STI co-infections, etc.).

We also include phenomenological heterogeneity in the effective mixing rates to account for behavioural change as the epidemic progresses. We set 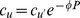 and 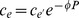 where 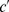 is the baseline effective mixing rate and 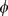 the strength of the behavioural response [25]. The range of values for the phenomenological parameter 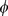 (Table 1) were chosen after fitting both prevalence trajectories and observed behaviour changes (for the latter, we assumed change in reported condom usage from DHS data was a fair proxy for behaviour change) for sub-Sahara African countries where such data were available.

**Table 1:** Ranges of model parameters used in the latin hypercube sampling.

Parameter	Range	Source
Death rate *µ*	1/60–1/40	UN
Disease-induced death rate *α*	1/16–1/4	[30], [31]
Couple formation rate *m*	1/20–1/5	Inferred from DHS
Couple dissolution rate *δ*	1/30–1/10	Inferred from DHS
Effective uncoupled contact rate *c_u_*	0.05–0.25	Assumption
Effective within-couple contact rate within serodiscordant *c_w_*	0.05–0.25	[11], [15], [22], [26]–[28]
Relative contact rate extra-couple *c_e_*/*c_w_*	0.01–1	Assumption
Phenomenological decay *φ*	2–7	Inferred from DHS

These ranges are to represent realistic values for Sub-Saharan Africa. Unit of all rates is per year.

We assume that individuals mix homogeneously when interacting with individuals other than their stable partners; thus 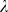 is given by the proportion of mixing in the non-couple pool that is accounted for by infectious individuals:
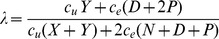
(2)


Within-couple transmission also has an implicit prevalence term: within-couple prevalence is 0 for concordant negative couples, and 1 for the susceptible individual in a serodiscordant couple.

The dynamical terms for disease transmission can now be calculated. The flow of singles from 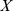 to 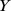 is 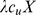. A concordant negative couple (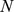) moves to 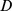 if either partner is infected, so this flow is 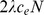. Couples move from 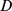 to 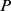 when the susceptible partner is infected from the mixing pool or by the infectious partner, that is a flow of 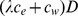.

### Recruitment and Death

A couple is dissolved when either partner dies. This happens at rate 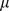 for susceptible individuals and at rate 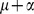 for infectious individuals. Thus, concordant couples are dissolved by death at rate 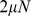 and 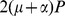, respectively, while serodiscordant are dissolved at rate 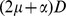. Surviving individuals are distributed to 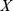 and 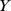. 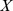 experiences a recruitment rate of 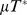 and a death rate 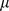. 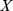 also increases when either partner of a sero-negative couple dies, or when the infected partner of a serodiscordant couple (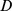) dies. Hence, 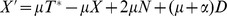. Similarly, 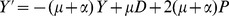.

### Combined Dynamics

Adding all the components above, the population dynamics are given by:
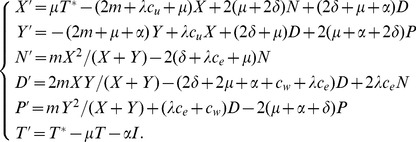
(3)


The global incidence is 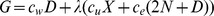, the first term being the incidence from within serodiscordant couples.

### Relative Incidences

The main outcomes studied here are the relative contribution of transmission to the global incidence from either uncoupled individuals or serodiscordant couples. We call 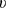 the proportion of global incidence due to transmission to uncoupled individuals and 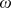 the proportion due to within-couple transmission. Hence, using the model notation, we have:
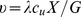
(4)

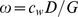
(5)


The importance of within-couple transmission has been measured in several different ways. For example [2], [5] estimated what we call 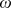 – the proportion of *all* infections that are due to within-couple transmission. Another study [7] considered all transmissions *to* couples that were infected by each of the three routes: pre-couple formation and within or outside couple transmission. Here, we use another ratio which is more appropriate to our model and define 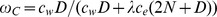 as the proportion of these infections that are due to within-couple transmission when only coupled individuals are accounted for.

**Figure 2:**
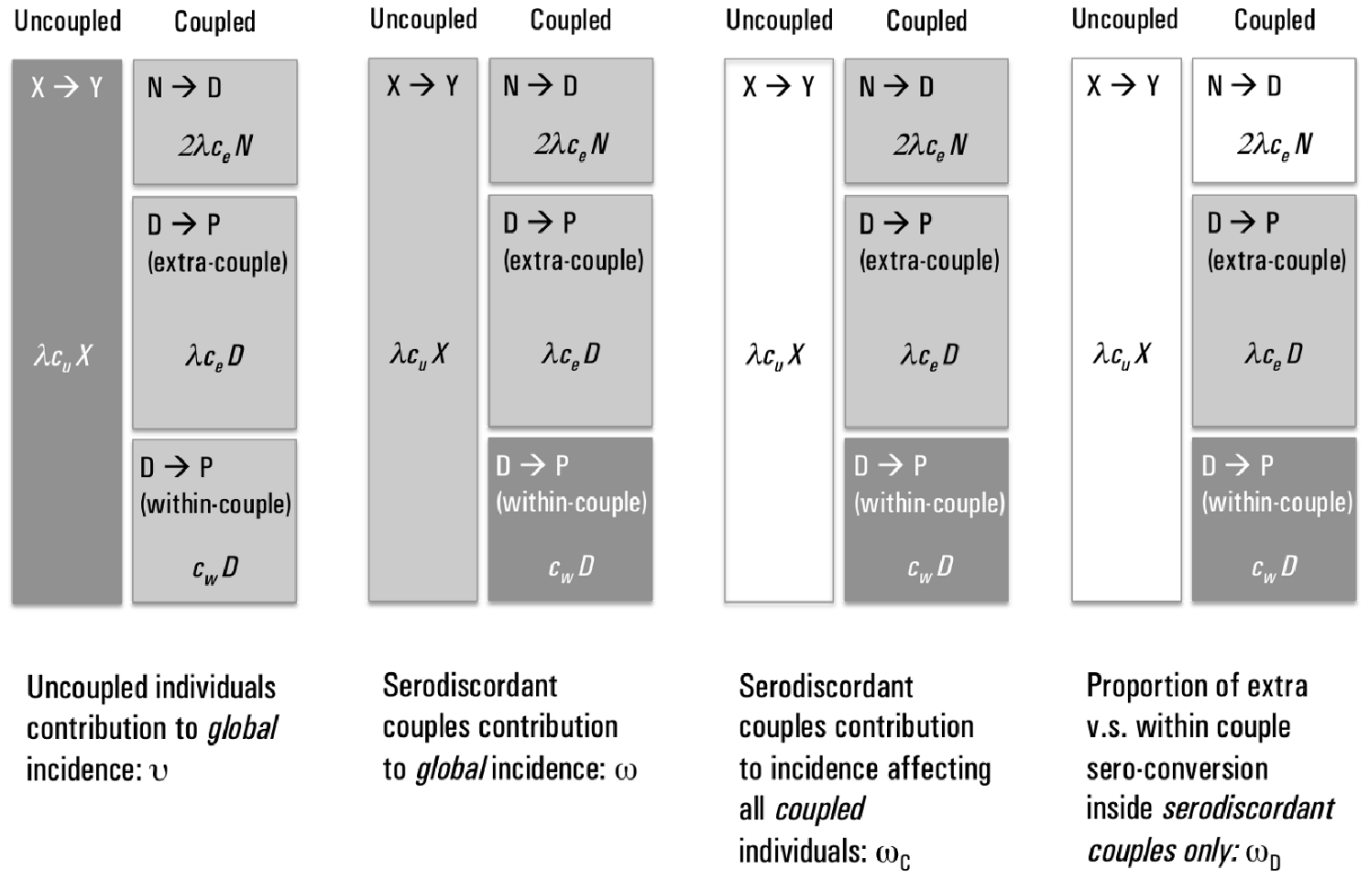
Incidence proportions. Different measures of the proportion of within-couple transmission have been used in the past, this figure illustrates the measures discussed here. Each panel graphically represents how the incidence proportion is calculated: dark shaded compartment divided by all non-white compartments. Each compartment represent a transmission route. The proportion of new HIV infections due to uncoupled individuals (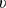) is illustrated in the left panel. The next three panels show the different definitions of the proportion of within-couple transmission calculated as a fraction of other transmission components: global transmission (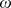, all compartments, middle left panel); transmission to coupled individuals (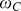, middle right panel); or transmission within serodiscordant couples, (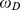, right panel).

Finally, the model in [9] was restricted to the proportion of infections transmitted within serodiscordant couples only; we call this quantity 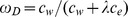. Figure 2 illustrates the difference between these ratios.

We measure all 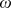
s and 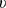 at the time horizon of our simulations, set at 40 years. Numerical simulations indicate that results are not sensitive to this choice as these ratios tend to converge quickly to their equilibrium values (see File S1).

### Serodiscordance Statistic

We also create a unitless measure of serodiscordance to compare with the proportion 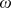. If no transmission happened in couples (or if dissolution dynamics were very fast), we would expect the proportion of all couples that are serodiscordant to be 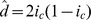, where 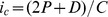 is the proportion of all coupled individuals who are infectious and 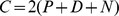 is the number of individuals living in a stable couple. We can then compare this expectation to the observed proportion of serodiscordant couples 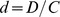, and define a unitless serodiscordance statistic 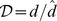 that measures how serodiscordant the population is compared to this null model.

### Numerical Simulations

Unfortunately, even this simplified model does not provide simple analytic insights when both partnership dynamics and HIV-induced mortality are included. We therefore used numerical simulations to explore a broad range of plausible parameters.

### Latin Hypercube Sampling

We perform latin hypercube sampling on the model parameters and examine how measures of prevalence, discordance and within-couple transmission are distributed, and how they are correlated with parameters. Every parameter 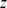 was assigned a range between 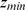 and 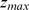 and 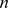 values are equally spaced on the log scale from 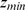 to 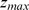 (i.e. the *ratio* between successive values is the same, see File S1 for more details).

### Parameter Ranges

Table 1 summarizes the ranges used for all model parameters. The parameter ranges are chosen to reflect demography and heterosexual HIV transmission in Sub-Saharan Africa; details are described in File S2.

The natural death rate 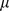 was chosen to reflect the range of life expectancies found in Sub-Saharan Africa and also the fact we are considering sexually active individuals (assumed over 15 years old, see File S2).

The disease-induced death rate is relatively well documented and we chose a range consistent with published studies (see File S2).

Couple formation and dissolution rates (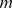 and 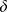) are uncertain. However, our model gives an analytical relationship between the coupled population at the disease-free equilibrium (DFE) and the parameters 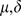 and 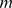 (see File S1 for details). Hence, we chose to calibrate 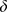 and 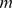 to the DHS data of proportion of coupled individuals while also yielding realistic distributions of relationship durations (see File S2).

The susceptible groups 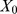 and 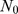 are set at the DFE of our model. A small amount of infectious individuals is introduced to start the epidemic (see File S1 for details).

The hazard of within-couple transmission 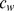 has been estimated by numerous serodiscordant couple cohort studies (see for example [11], [15], [22], [26]–[28]) and our range was chosen to reflect these findings. Little information is available about the pool mixing rates, 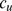 and 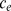. We decided to use the same range for 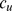 as for 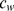 – in other words, we explore the same ranges of sexual contact rates for uncoupled individuals mixing with uncoupled individuals as for individuals with their stable partners. We assumed the effective extra-couple contact rate 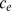 is less than the within-couple rate 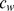 (also recall that effective contact rates are multiplied by prevalence to yield transmission hazards). We therefore allowed the ratio 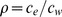 to vary between 0.01 and 1.

### Sensitivity Analysis

In order to conveniently assess the main drivers of HIV incidence as well as discordance in our model, a sensitivity analysis was performed. Details of the methodology are given in File S1.

## Results

Simulations shown hereafter were run with 10,000 samples. The time horizon for the simulations was set at 40 years.

### Relative Incidences

**Figure 3:**
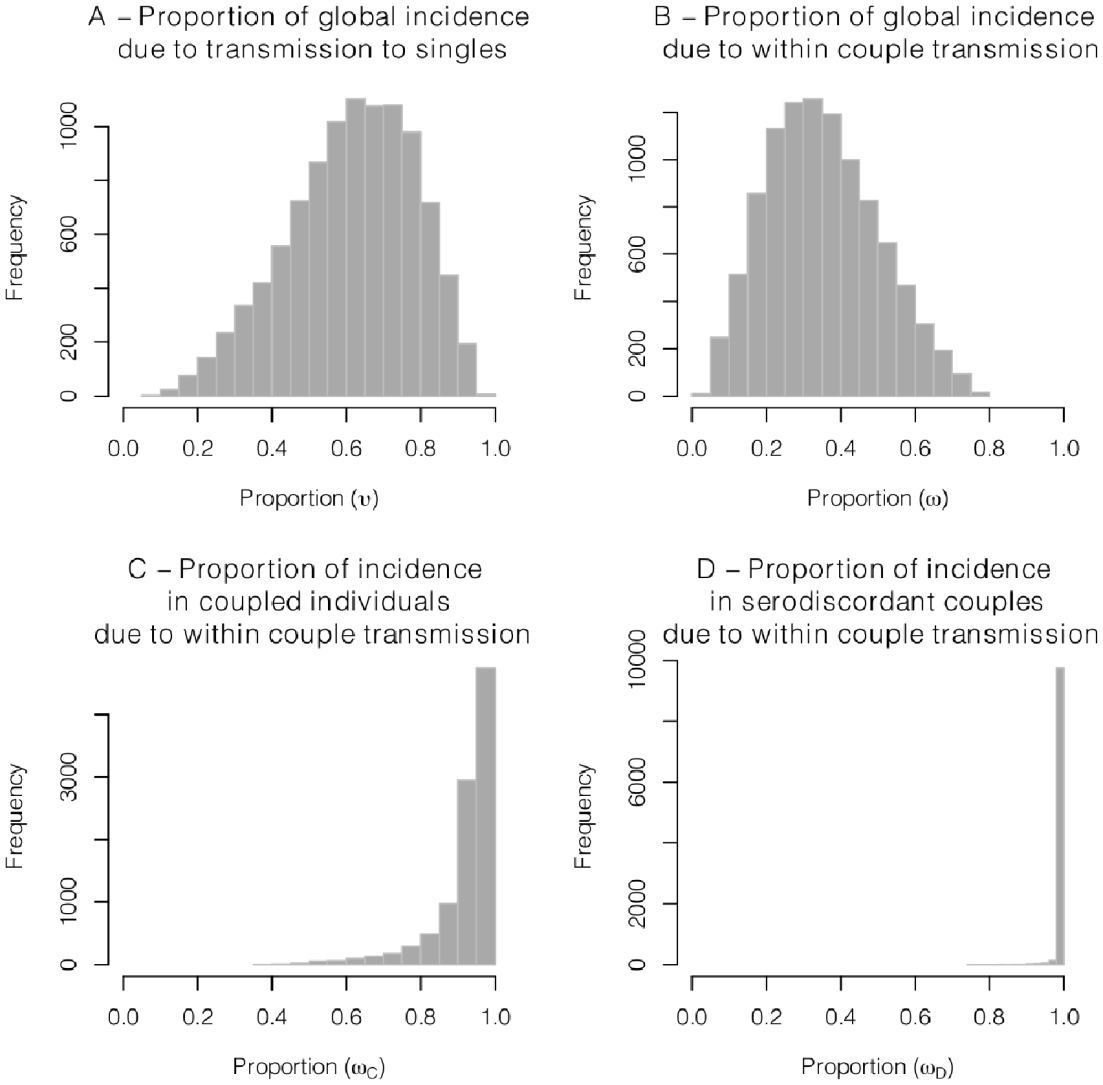
Simulated incidence proportions. Histograms of the transmissions proportions occurring in uncoupled and serodiscordant couples at maturity from 10,000 latin hypercube samplings. Ranges are specified in Table 1. When compared to the total incidence at the whole population level, transmission to singles accounts for a large proportion of all cases (panel A) whereas within-couple transmission accounts for a low to moderate proportion (panel B). But when compared to the incidence occurring only among all coupled individuals (discordant or not), the share of within-couple transmissions is much higher (panels C and D). Hence a low importance of within-couple transmission at the whole population level is consistent with high importance of this route of transmission limited to the coupled population.

Figure 3 shows various measures of the importance of singles and serodiscordant couples to HIV incidence at the time horizon of our simulations. These quantities come to equilibrium relatively quickly in our model, and so the values here will be very close to equilibrium values.

In the parameter space explored in Table 1, Figure 3 panel A shows that at equilibrium HIV incidence is in most cases primarily driven by cases due to transmissions between singles, our simulations giving a median value of 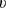 at 0.62 (95% of all simulations fall between 0.26 and 0.88).

Panel B shows that 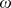, the equilibrium contribution from transmission within serodiscordant couples at the *whole population* level, is mostly constrained to relatively low levels (median is 0.33 and 95% of all simulations fall between 0.10 and 0.67 ) as shown in Figure 3 panel B. In other words, it is unlikely for mature epidemics to be driven primarily by transmission within stable couples.

Importantly, low importance of within-couple transmission in the *whole* population (low values of 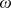) is consistent with high values among *coupled* individuals (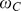, panel C) and particularly among serodiscordant couples (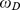, panel D). In particular, our relatively low values for 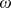 are consistent with the country-specific estimates of 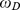 from [9].

### Long-term Effects of Transmission Routes

**Figure 4:**
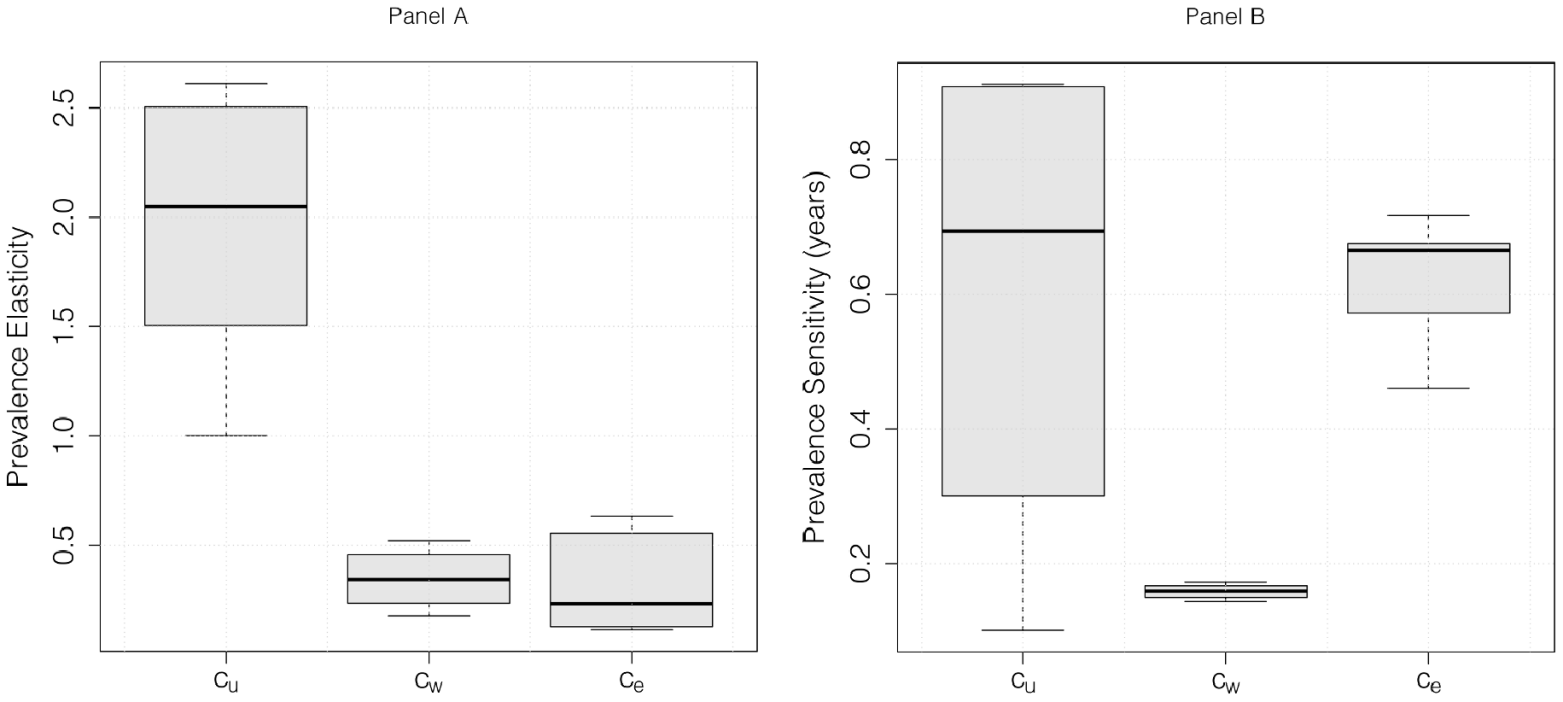
Prevalence sensitivities. Left panel shows the elasticities (unitless) of overall HIV prevalence to the three effective mixing rates (proportional change of prevalence for a given proportional change of 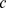, that is 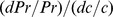, with 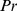 the prevalence). Right panel shows the sensitivities (absolute change of prevalence for a given absolute change of 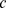, that is 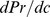. Units in years). See main text for interpretations.

We further elucidate the “importance” of different routes of transmission by asking what would happen to long-term (i.e. equilibrium) HIV prevalence if mixing rates were to change. Figure 4, panel A shows that a proportional change in the mixing rate of uncoupled individuals 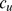 is expected to have a much larger effect on the epidemic than the same proportional change in either 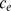 or 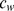.

The reasons why the other two mixing rates have less proportional effect on prevalence are different for 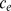 and 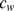. In the case of extra-couple contact 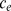, panel B shows that if we consider *absolute* changes in mixing rate, the effects of changes in 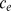 and 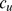 are similar. Thus, the relatively low proportional effect of 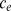 is due to our assumptions: we always assume that 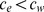, and over most of our parameter range it is much less, while we let 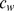 and 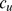 vary over the same range. When 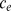 is small, proportional changes in 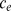 will have relatively little effect.

In contrast, even absolute changes in the within-couple effective contact rate 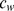 have a relatively small effect on prevalence. This is due to the fact that the serodiscordant population to which 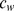 applies (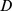) is much smaller than the uncoupled (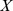) and coupled (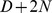) susceptible individuals. Our model initially fits the proportion of coupled individuals (infected or not) to actual demographic data (File S2), and the proportion of discordant couples that emerges from our model remains relatively low throughout our simulations. This in turn has two causes: relatively few people are infected with HIV most of the time; and people with HIV-infected partners are relatively less likely to be susceptible, because they are likely to have been infected by their partners already.

Hence, our result on the importance of uncoupled mixing rates in driving prevalence is underpinned by uncoupled individuals constituting a large proportion of the sexually active population (fitted to actual data), an extra-couple mixing rate (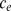) up to 2 orders of magnitude lower than the one of uncoupled (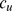) and a proportion of discordant couples that remain low throughout our simulations (File S1).

### Serodiscordance Statistic and Backward Interpretation

**Figure 5:**
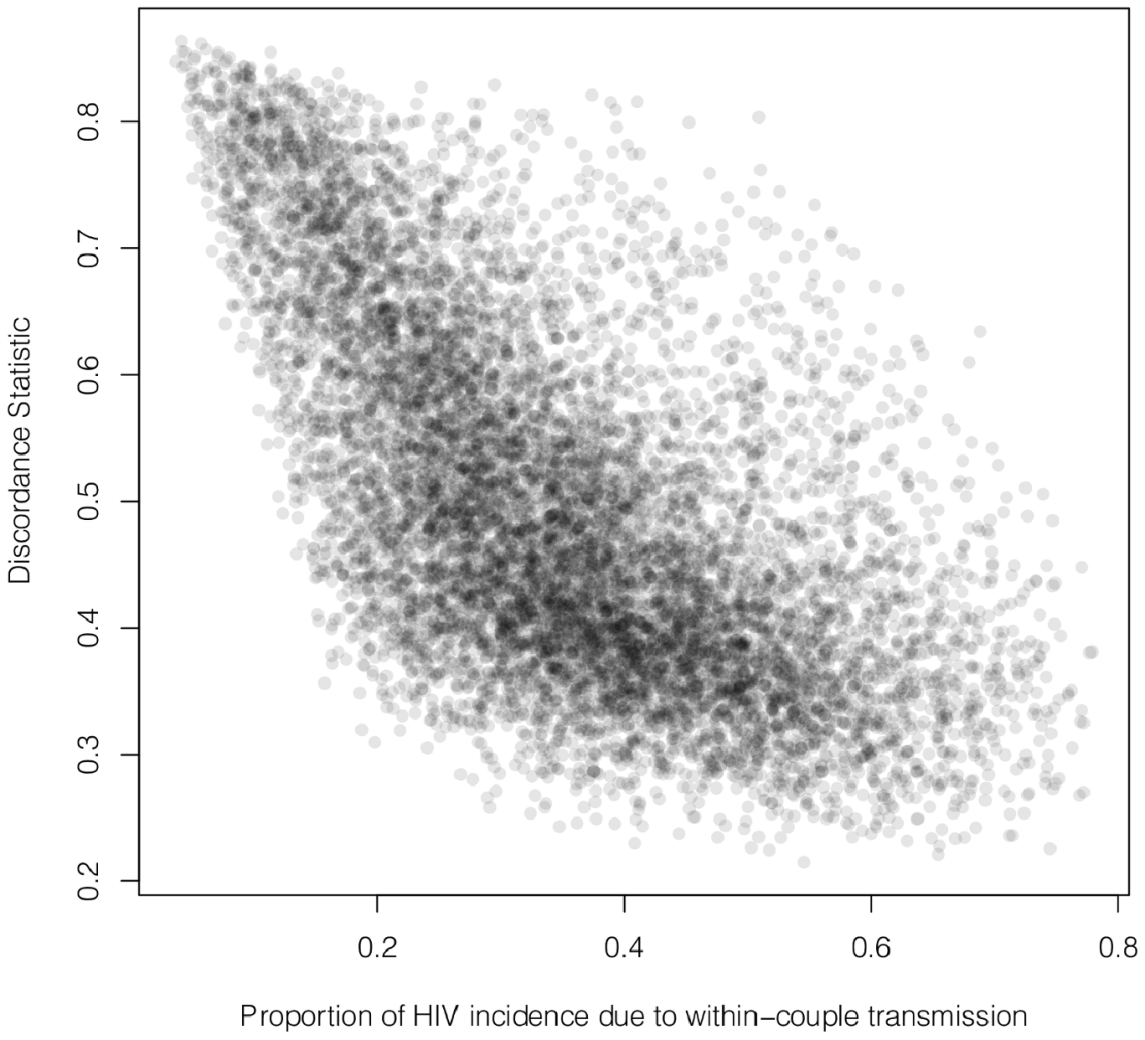
Discordant statistic and within-couple transmission contribution. The discordance statistic 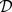 as a function of the contribution of within-couple transmission to the global incidence (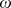). Our 10,000 simulations run with parameters sampled from realistic ranges (Table 1) show a negative relationship, suggesting that for a given HIV prevalence in the whole population, the observed discordance (measured with 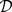) may be a signature of the importance of within-couple transmission.

Another interesting result from the model is the negative relationship between the level of serodiscordance in the whole population (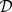) and the contribution of within-couple transmission to global incidence (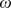) as illustrated in Figure 5. Hence, at a given prevalence, a high observed discordance is associated with a relatively low contribution of within-couple transmission to the total incidence.

**Figure 6:**
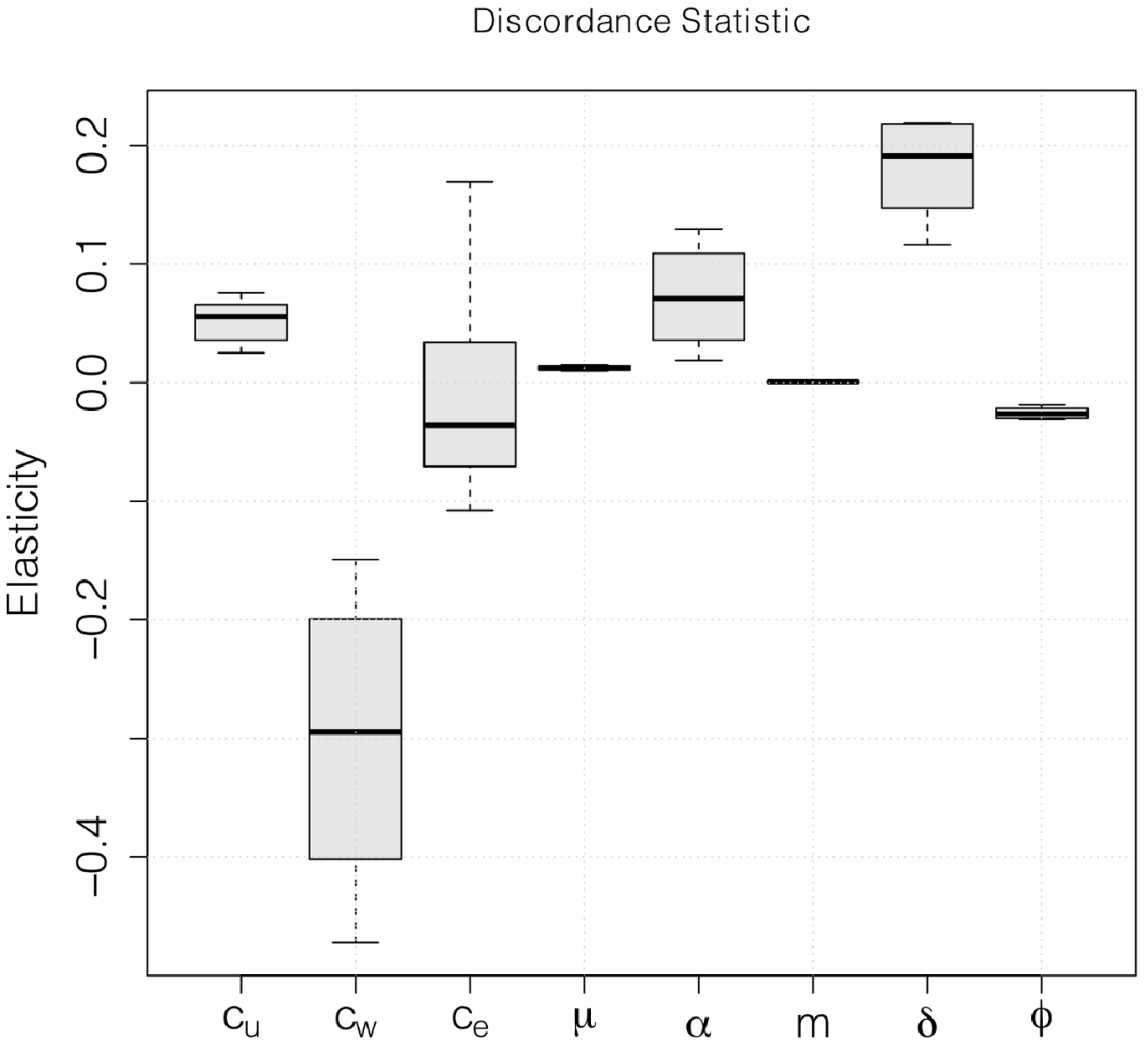
Discordant statistic elasticities. Elasticities of the discordance statistic 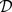 to all model parameters. The relatively large negative elasticity of the mixing rate within discordant couples, 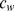, shows its negative relationship with 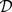.

Furthermore, results in Figure 6 show this same level of discordance (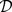) exhibits a strong negative correlation with the within-couple transmission rate (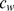).

These results give more support to the “backward” interpretation, where – for a given prevalence – high observed serodiscordance is likely to be a signature of non-couple routes of transmission and their interactions with the partner switching dynamics.

## Discussion

Identifying the main factors that drive transmission of a sexually transmissible disease is key to designing effective interventions and, in the context presented here, to allocating resources between couple-based and population-based interventions.

The importance of non-couple versus couple-based transmission, and more specifically the role of serodiscordant couples in HIV transmission remains controversial [2]–[9]. Using a simple dynamical model, we explored a plausible parameter space for HIV transmission in Sub-Saharan Africa, and found that prevalence was mainly driven by the mixing rate of uncoupled individuals. Furthermore, within-couple transmission had low to moderate importance at the whole population level in transmitting HIV under all combinations of our parameters (Figure 3). Simultaneously, we found that within-couple transmission contributed to the majority of secondary infections within serodiscordant couples. Thus, estimates of a high importance of within-couple transmission at the level of the sub-population of serodiscordant couples [9] are consistent with estimates of relatively low importance of this route of transmission in the whole population [3], [5]–[7].

Our model also sheds light on what inferences can be made from measured levels of serodiscordance. We introduced a unitless index of discordance (the proportion of couples which are discordant, relative to a random expectation), and found negative correlations between discordance and both the within-couple transmission effective mixing rate 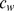 and the proportion of total HIV incidence due to within-couple transmission, 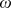. This lends credence to what we have called the “backward” interpretation – that for a given prevalence higher levels of discordance suggest a greater role of non-couple routes of transmission and their interactions with the partner switching dynamics.

To efficiently explore a poorly understood parameter space, our model made a large number of simplifying assumptions. We did not include gender asymmetries – however, there is evidence that these are not very strong [7], [21], [22]. We model a form of concurrency by allowing partners to have outside relationships, but do not explicitly model concurrent, stable relationships, which may also be an important factor.

We also assume that the transmission rate is constant throughout the natural history of disease; in particular, we do not model the acute phase of increased HIV infectiousness 6 to 8 weeks after HIV acquisition [29]. This effect could either increase within-couple transmission (when one member of a susceptible couple is infected via extra-couple contact) or decrease it (when infection occurs well before couple formation). To some extent, these two effects should balance out.

Our model also assumes that all mixing between non-stable partners only occurs as one-off interactions rather than as longer sustained interactions. This simplification is commonly used in models of sexually-transmitted diseases. Allowing non-stable interactions to involve multiple contacts would primarily affect model dynamics by causing some individuals to spend more time with infected individuals and others to spend more time with uninfected individuals, thereby creating a more heterogeneous distribution of risk.

Future work should investigate the robustness of our conclusions when more types of heterogeneity – such as the greater infectiousness of the acute phase, gender asymmetries, super-spreader groups, etc – are included. We note that our analysis provides a simple framework from which to analyze the fundamental forces driving incidence among coupled and uncoupled individuals, and that analyses of more complex models will require great care in order to clearly disentangle the causal dynamical processes.

In conclusion, our results provide further evidence that transmission within couples, extra-couple transmission and transmission to uncoupled individuals are all likely to be important in sustaining heterosexual HIV transmission in Sub-Saharan Africa. Infections of uncoupled individuals, in particular, were identified in our model as a key driver of long-term HIV prevalence and thus should be appropriately targeted by interventions.

## Supporting Information

File S1 **Model Details.**
(PDF)

File S2 **Parameters Sources.**
(PDF)

File S3 **Source Code.**
(PDF)
