# Supplementary material for: HIV Sexual Transmission Is Predominantly Driven by Single Individuals Rather than Discordant Couples: A Model-Based Approach
Source: PLoS One. 2013 Dec 20;8(12):e82906. doi: 10.1371/journal.pone.0082906 (PMC3869741; doi:10.1371/journal.pone.0082906)
Supplement: File S1 — Model Details. (PDF) [file pone.0082906.s001.pdf]

Supporting Information: File S1 – Model Details

1 Couple formation

The way couples form is assumed to follow a binomial distribution. If $z \in \{0, 1, 2\}$ is the number of susceptible individual(s) just before a couple formation, then we assume $z \sim \text{Binom}(2, p_s)$ with $p_s = X/(X+Y)$ the probability that an uncoupled individual is susceptible. Thus

$$p(z = k) = \binom{2}{k} p_s^k (1 - p_s)^{2-k} \tag{1}$$

Hence, we have the following proportions:

- Proportion of couple formation within X is $\frac{X^2}{(X+Y)^2}$
- Proportion of couple formation within Y is $\frac{Y^2}{(X+Y)^2}$
- Proportion of couple formation between X and Y is $\frac{2XY}{(X+Y)^2}$

2 Equilibrium in simulations

In order to check that our simulations are reasonably close to the equilibrium, we monitor the derivative of prevalence at the horizon of the simulation (set at 40 years). As shown in Figure S1, for 10,000 simulations where the parameters have the same constraints as the LHS, the mean value of the normalized derivatives ($dX/X/dt$) for all group is small, comforting we are close to an equilibrium in most of our simulations.

3 Disease free equilibrium

At the disease free equilibrium we have $Y = D = P = 0$ and $X' = N' = 0$. The system (**??**) becomes

$$\begin{cases} X' = \mu T^* - (2m + \mu)X + 2(\mu + 2\delta)N \\ N' = mX - 2(\delta + \mu)N \end{cases} \tag{2}$$

which can easily be solved, giving the proportion of uncoupled and coupled individuals

$$2N^* = \mu T^* \left[(2m + \mu)(\delta + \mu)/m - (\mu + 2\delta)\right]^{-1}$$

$$X^* = \frac{(\delta + \mu)}{m} 2N^*$$

Because $T^* = X^* + 2N^*$ we can substitute,

$$X^* = \sigma T^* \tag{3}$$

$$2N^* = (1 - \sigma)T^* \tag{4}$$

with

$$\sigma = \frac{\mu + \delta}{\mu + \delta + m} \tag{5}$$

being the proportion of single at DFE when the recruitment rate balances the death rate.

4 Initial infectious individuals

If U_0 (resp. C_0) is the initial uncoupled (resp. coupled) population, we introduce a small amount ϵ of infectious individuals such that $Y_0 = \epsilon U$ and $X_0 = (1-\epsilon)U_0$. Similarly, $P_0 = \epsilon^2 C_0$, $N_0 = (1-\epsilon)^2 C_0$ and $D_0 = 2\epsilon(1-\epsilon)C_0$. The value for ϵ does not affect significantly our results as long as it is sufficiently small. We chose $\epsilon = 0.01$.

5 Latin hypercube sampling

Every parameter z was attributed a range between z_{min} and z_{max}. Then, this range is partitioned log-proportionally

$$z_i = \exp\left(\log(z_{min}) + [\log(z_{max}) - \log(z_{min})]\frac{i-1}{n-1}\right) \tag{6}$$

with n the total number of samplings and $i = 1,..,n$. The (ordered) partition $[z_1,...,z_n]$ is then randomly shuffled, independently for each parameter, leading to a permuted vector of values $[\zeta_0, \zeta_1, ..., \zeta_n]$. Assuming there are K model parameters to be sampled, we have a $n \times K$ sampling matrix, noted $(\zeta_i^k)_{i=1..n,\, k=1..K}$. The ith row ζ_i of this matrix represents the ith simulation run with the set of K parameters randomly assigned from the partitions.

6 Discordance Statistic

Figure S2 shows the distribution of the discordance statistic $\mathcal{D}$ from the 10,000 samples of the LHS.

7 Sensitivity Analysis

The methodology to calculate the *elasticity* of a response variable (e.g. prevalence) Z to the model parameters is the following. Let p be a parameter on which the elasticities will be calculated. The latin hypercube sampling (LHS) range for p is partitioned in n values: $p_1, p_2, ..., p_n$. For the first LHS run, p will be fixed at p_1 and all other parameters will be sampled in their respective predefined ranges. For the first LHS run, the average of all Z (defined as $\langle Z\rangle_1$) is calculated. The LHS run is repeated n times and we calculate $e_{p,i}$, the elasticity of parameter p between values p_{i-1} and p_i, as

$$e_{p,i} = \frac{\log\langle Z\rangle_i - \log\langle Z\rangle_{i-1}}{\log(p_i) - \log(p_{i-1})} \tag{7}$$

For a given parameter p, there are $(n-1)$ such sensitivities. Then, we define $e_p = (\sum_i e_{p,i})/(n-1)$, the averaged value of these elasticities. This is this quantity that is reported in the main text. A positive (resp. negative) elasticity on Z means increasing the parameter moves the distribution of Z to larger (resp. smaller) values.

For the *sensitivities*, we apply the same methodology, except that now, the sensitivity formula for the parameter p between values p_{i-1} and p_i is

$$s_{p,i} = \frac{\langle Z\rangle_i - \langle Z\rangle_{i-1}}{p_i - p_{i-1}} \tag{8}$$

7.0.1 Elasticity of prevalence with respect to all parameters

Figure S3 shows the elasticity of the overall HIV prevalence with respect to all model parameters.

7.0.2 Sensitivity of ω_C and ω_D

Before calculating the sensitivity, let us recall the formula defining the proportion of incidence due to within couple transmission:

$$\omega_C = \frac{c_w}{c_w + \lambda c_e(1 + 2N/D)} \text{ and } \omega_D = \frac{c_w}{c_w + \lambda c_e}$$

Note also that increasing c_w decreases D and increases P (the discordant state is more transient) and prevalence –in particular the one in the mixing pool λ – increases too. So we can expect two offsetting effects on ω when increasing c_w, and this is what we can observe in Figure S4 where the sensitivities ($d\omega/dc$) to the effective contact rates are plotted. These plots are comparable with the plot in Figure 4 in the main text, right panel.

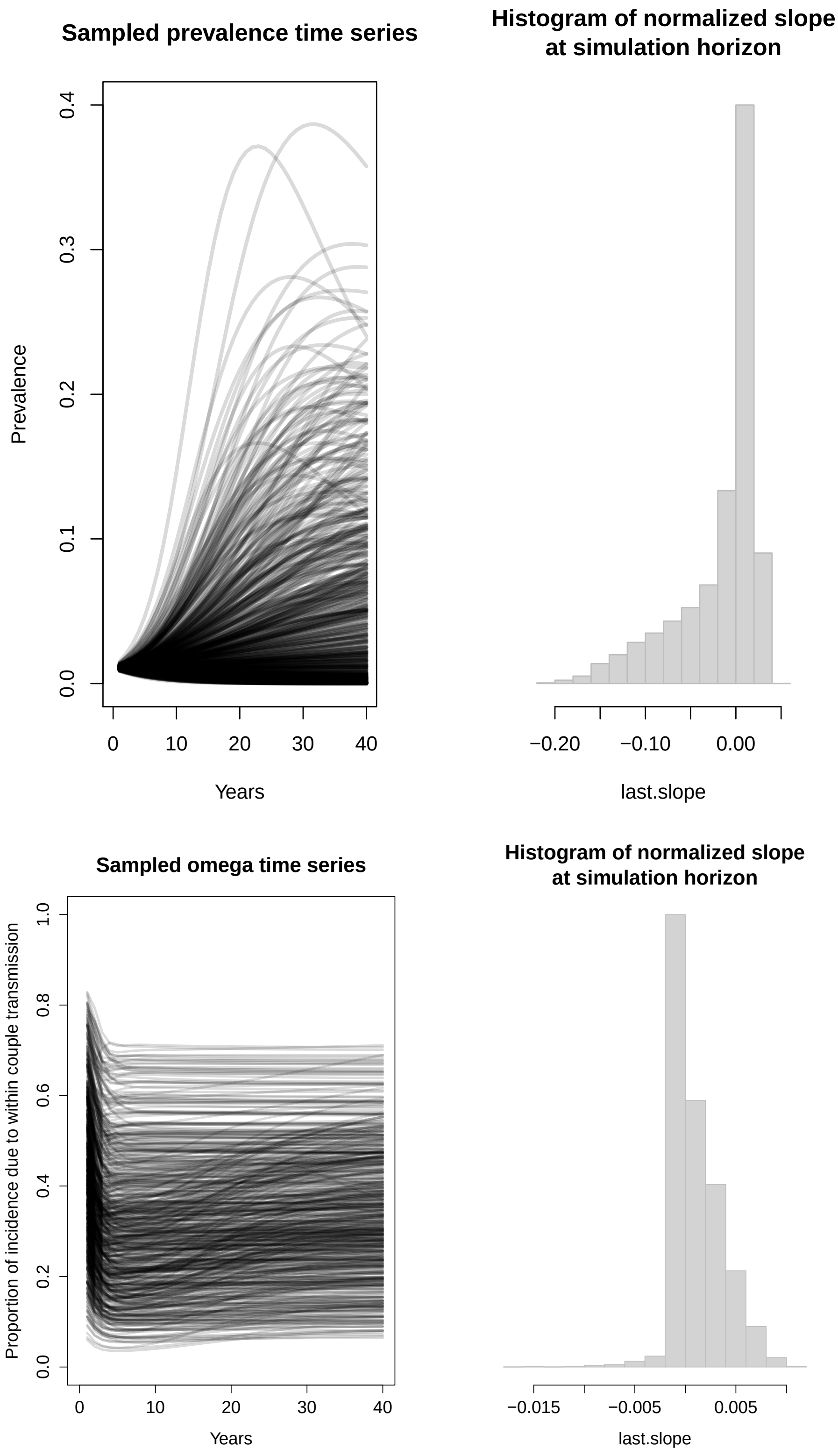

Figure S1. Prevalence and incidence proportion at time horizon. Sampled prevalence time series and its normalized derivatives at time horizon (year^{-1}) from 10,000 simulations. For both total prevalence and the proportion of global incidence due to within-couple transmission (ω), most of the simulated values at time horizon have reached an equilibrium.

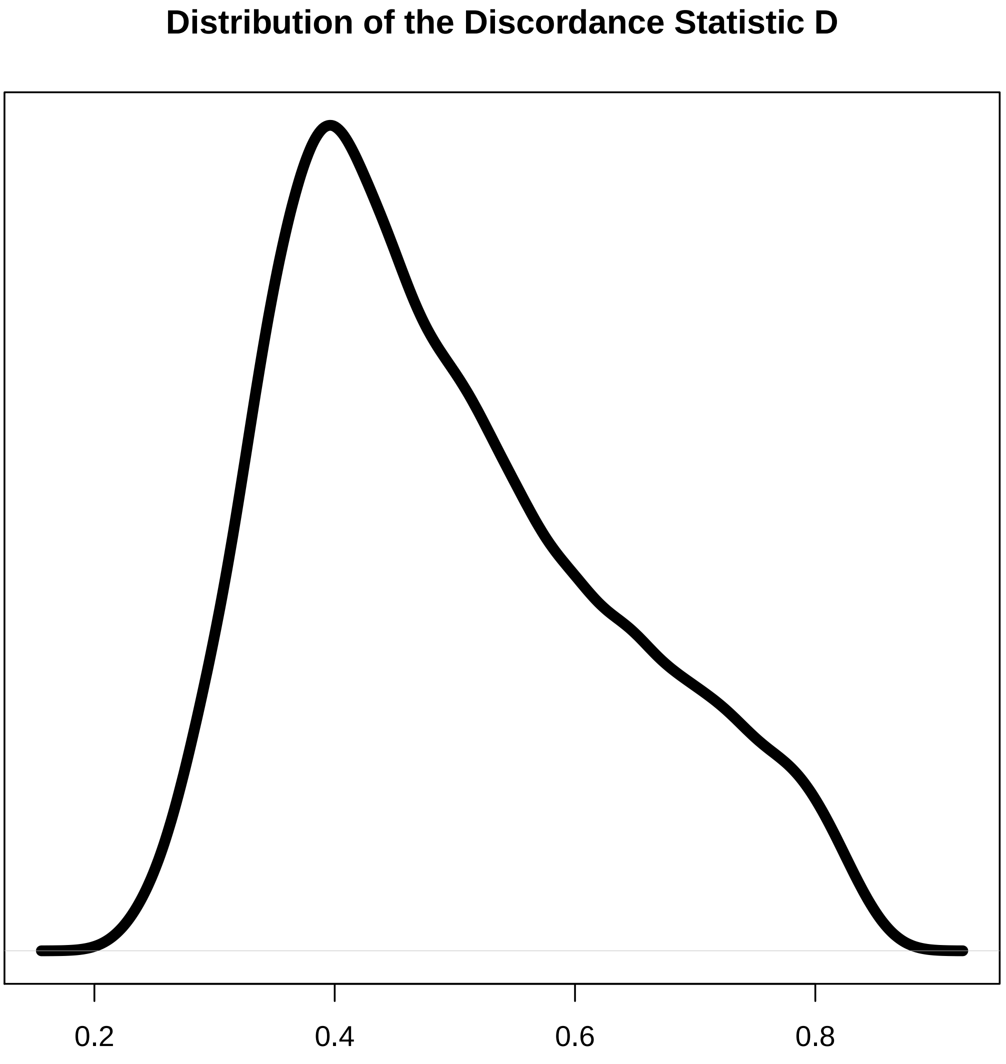

Figure S2. Distribution of the discordance statistic. Distribution of the discordance statistic $\mathcal{D}$ from the 10,000 simulations with parameters sampled in the range of Table 1 (main text).

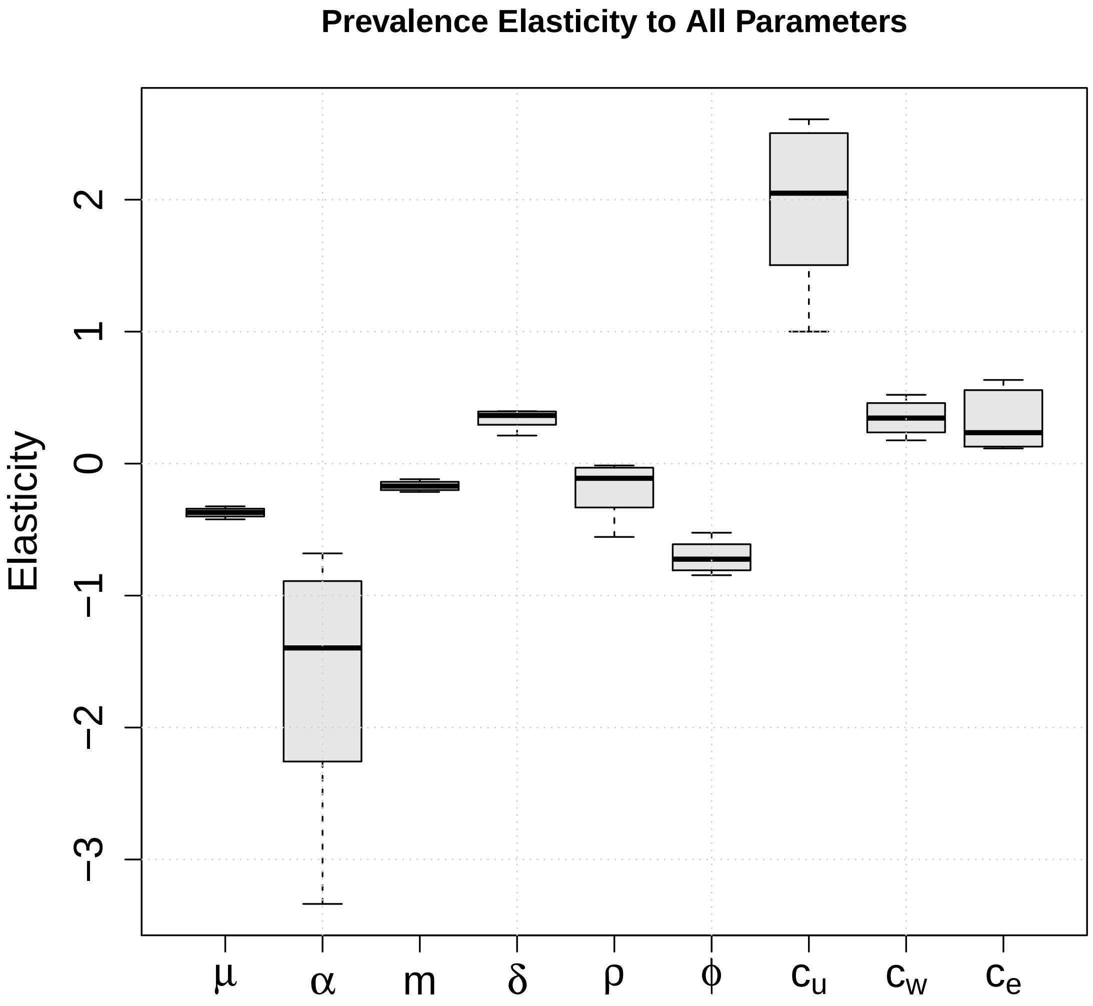

Figure S3. Prevalence elasticities. Elasticity of prevalence with respect to all parameters

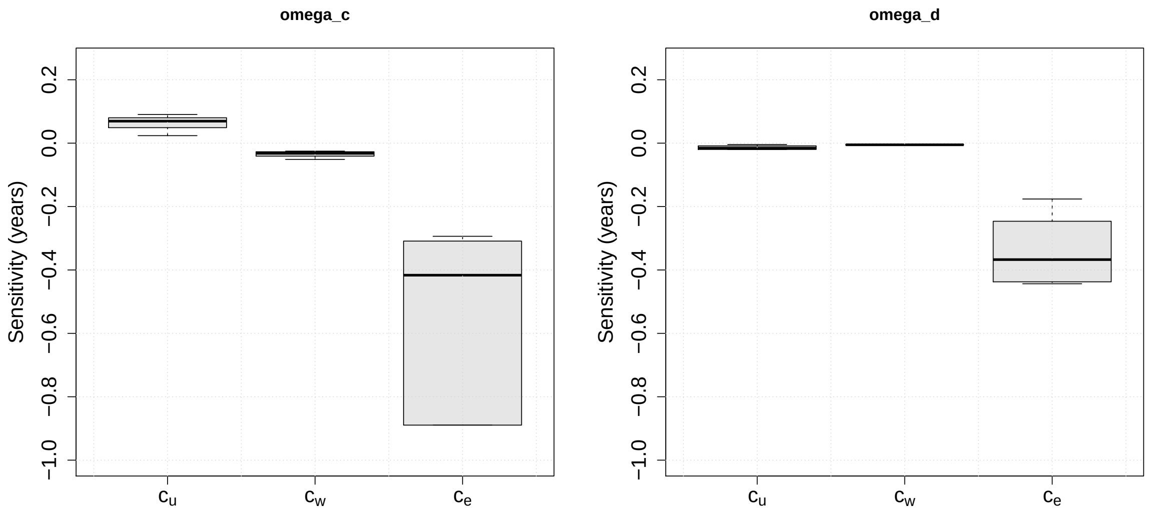

Figure S4. Incidence proportion sensitivities. Sensitivities of ω_C and ω_D with respect to the effective contact rates
