# Supplementary material for: HIV Sexual Transmission Is Predominantly Driven by Single Individuals Rather than Discordant Couples: A Model-Based Approach
Source: PLoS One. 2013 Dec 20;8(12):e82906. doi: 10.1371/journal.pone.0082906 (PMC3869741; doi:10.1371/journal.pone.0082906)
Supplement: File S2 — Parameters Sources. (PDF) [file pone.0082906.s002.pdf]

Supporting Information: File S2 – Parameters Sources

Vital rates

Life expectancies at birth from all countries in sub-Saharan Africa were downloaded from the UN website (http://esa.un.org/unpd/wpp/Excel-Data/mortality.htm accessed Nov 1st, 2012). French territories, Mayotte and Reunion, were not taken into account. Figure S5 illustrates this data set.

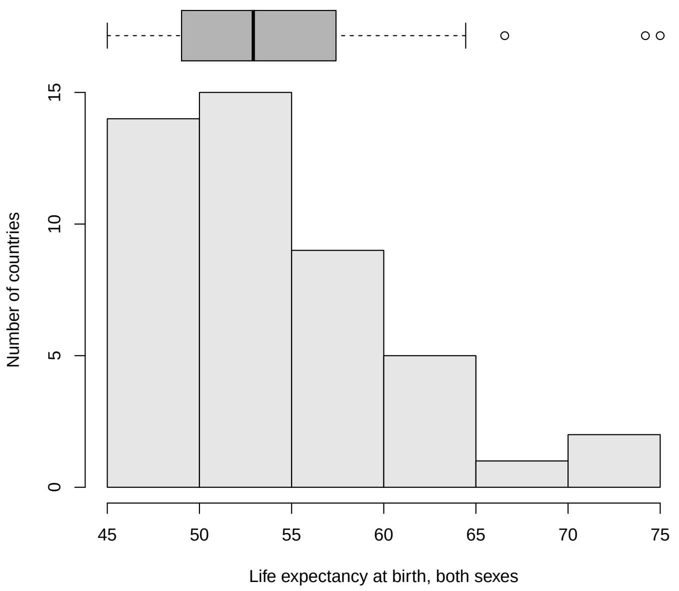

Figure S5. Life expectancies. Histogram of life expectancies at birth for both sexes from countries in sub-Saharan Africa. Source: UN.

HIV induced mortality rate

The HIV induced mortality rate range used in our study was based on mainly two previous studies [1, 2] before ART introduction. The latest [2] studied low and middle income countries, which are more similar to our sub-Saharan countries. than those in the older study [1], which focused on high-income countries. The findings of these two studies are summarized in Figure S6 where the 95% confidence intervals of the median survival time are shown for different cohorts.

Coupled population

We used the DHS data to assess the proportion of the population coupled. We gathered in the coupled category the individuals answering they were living as “Married” or “Living together”. The remaining answers (“Never married”, “Divorced”, “Widowed”, “Not living together” and “Missing”) were categorized as uncoupled. We looked at all countries from sub-Saharan Africa over all the years the DHS surveys were available as of November 1st, 2012. Results are plotted in Figure S7.

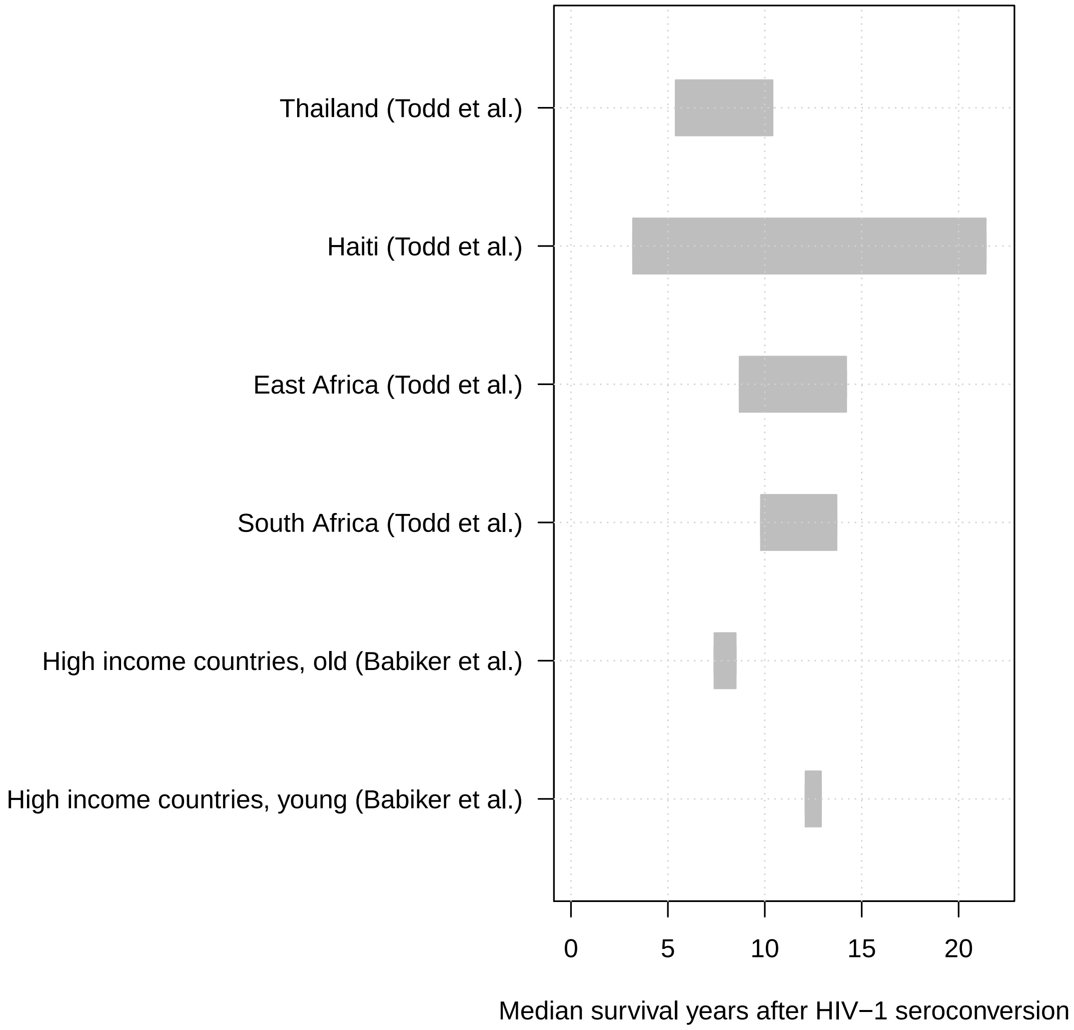

Figure S6. HIV survival. Survival times after HIV infection from [1, 2]. See text in section for more details.

The values from the disease free equilibrium (DFE) formulas (see section **??** below) were compared to actual data from sub-Saharan Africa countries in the DHS database. We sampled 1,000 values using the latin hypercube method and chose values for δ, m to fit reasonably well the distribution of $2N^*$ with the distribution of DHS data. As shown in Figure S8, we obtained a fair match.

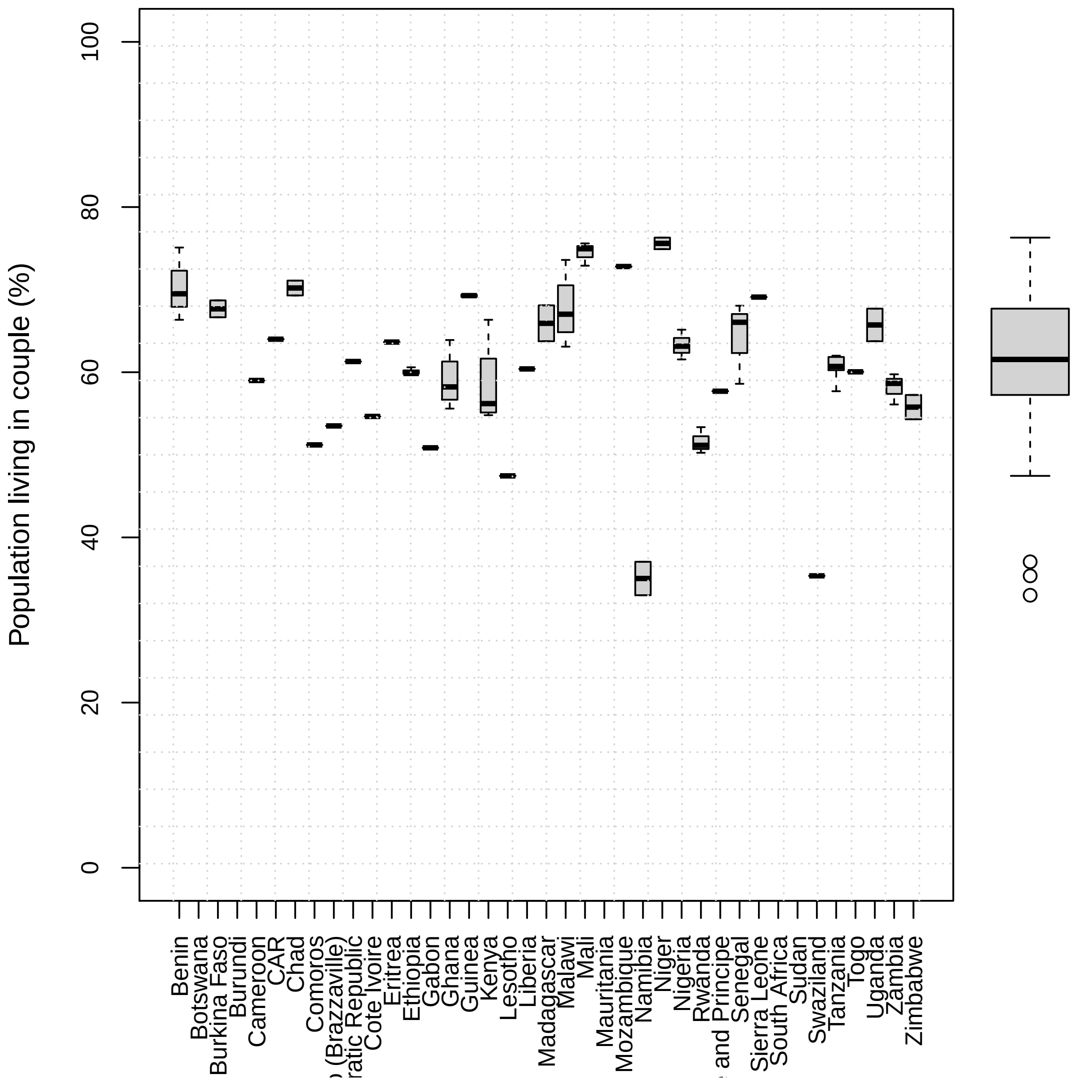

Figure S7. Coupled population. Percentage of the population living in coupled (source: DHS - http://www.measuredhs.com). If there is only one DHS survey for a given country, then the box plot is reduced a point. The large box plot on the right hand side agglomerates all values.

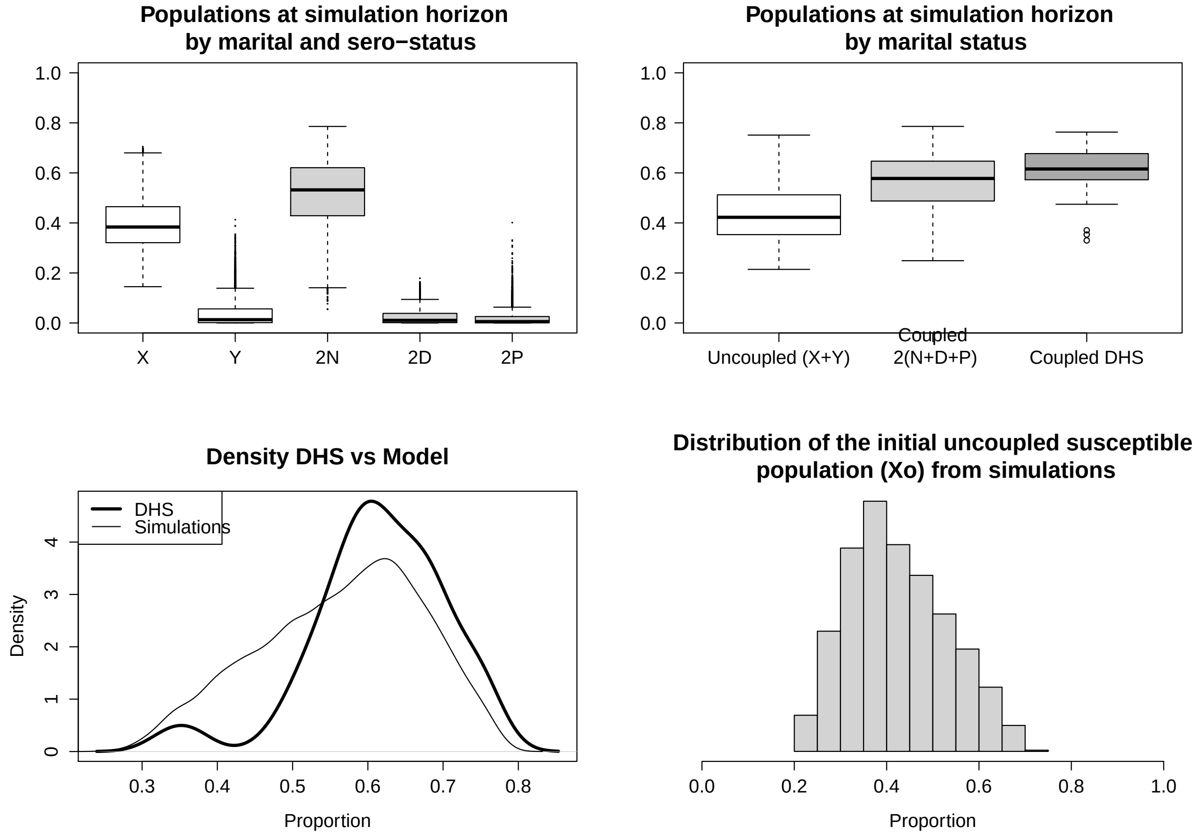

Figure S8. Simulated demographics. Top left panel: Proportions of populations at the time horizon of our 10,000 simulations by couple status and HIV serostatus. Top right panel: Proportions of population at the time horizon of our 10,000 simulations by couple status only and compared with DHS data of coupled individuals. Bottom left panel: Comparison of densities of coupled individuals between DHS data and the model simulations. Parameters range for couple dissolution and formation (δ and m) were chosen such that the model distribution of coupled individuals is similar to DHS data. Bottom right panel: initial distribution (from our 10,000 simulations) of the proportion of uncoupled individuals (X_0).

References

1. Babiker A, Darby S, De Angelis D, Kwart D, Porter K, et al. (2000) Time from HIV-1 seroconversion to AIDS and death before widespread use of highly-active antiretroviral therapy: a collaborative re-analysis. The Lancet 355: 1131–1137.

2. Todd J, Glynn J, Marston M, Lutalo T, Biraro S, et al. (2007) Time from HIV seroconversion to death: a collaborative analysis of eight studies in six low and middle-income countries before highly active antiretroviral therapy. AIDS 21: S55.
