# Supplementary material for: HIV Sexual Transmission Is Predominantly Driven by Single Individuals Rather than Discordant Couples: A Model-Based Approach
Source: PLoS One. 2013 Dec 20;8(12):e82906. doi: 10.1371/journal.pone.0082906 (PMC3869741; doi:10.1371/journal.pone.0082906)
Supplement: File S3 — Source Code. (PDF) [file pone.0082906.s003.pdf]

Supporting Information: File S3 – Source Code

The following URL gives access to the full computer code (C++ and R) used produce our findings:

http://lalashan.mcmaster.ca/theobio/Serodiscordance_Champredon_2013/index.php

You will need to have a C++ compiler (for example http://gcc.gnu.org) and the statistical software R (can be downloaded at http://www.r-project.org)

Reference

R Core Team (2012). R: A language and environment for statistical computing. R Foundation for Statistical Computing, Vienna, Austria. ISBN 3-900051-07-0, URL http://www.R-project.org/
